# Immunological profiles of the breast cancer microenvironment represented by tumor-infiltrating lymphocytes and PD-L1 expression

**DOI:** 10.1038/s41598-022-11578-x

**Published:** 2022-05-16

**Authors:** Toru Hanamura, Shigehisa Kitano, Hiroshi Kagamu, Makiko Yamashita, Mayako Terao, Banri Tsuda, Takuho Okamura, Nobue Kumaki, Katsuto Hozumi, Naoki Harada, Takayuki Iwamoto, Chikako Honda, Sasagu Kurozumi, Naoki Niikura

**Affiliations:** 1 Department of Breast Oncology, Tokai University School of Medicine, 143 Shimokasuya, Isehara-shi, Kanagawa Prefecture 259-1193 Japan; 2 Division of Cancer Immunotherapy Development, Center for Advanced Medical Development, The Cancer Institute Hospital of JFCR, 3-8-31, Ariake, Koto, Tokyo 135-8550 Japan; 3 Division of Respiratory Medicine, Saitama Medical University International Medical Center, 1397-1, Yamane, Hidaka-shi, Saitama Prefecture 350-1298 Japan; 4 Department of Pathology, Tokai University School of Medicine, 143 Shimokasuya, Isehara-shi, Kanagawa Prefecture 259-1193 Japan; 5 Department of Immunology, Tokai University School of Medicine, 143 Shimokasuya, Isehara-shi, Kanagawa Prefecture 259-1193 Japan; 6 Product Research Department, Chugai Pharmaceutical Co., Ltd., 200 Kajiwara, Kamakura-shi, Kanagawa Prefecture 247-8530 Japan; 7 Breast and Endocrine Surgery, Okayama University Hospital, 2-5-1 Shikata-cho, Kitaku, Okayama Prefecture 700-8558 Japan; 8 Department of General Surgical Science, Gunma University Graduate School of Medicine, 39-22, Showa-machi 3-chome, Maebashi-shi, Gunma Prefecture 371-8511 Japan; 9 Department of Breast Surgery, International University of Health and Welfare, 4-3, Kozunomori, Narita-shi, Chiba Prefecture 286-8686 Japan

**Keywords:** Cancer, Immunology, Biomarkers, Oncology

## Abstract

Tumor-infiltrating lymphocytes (TILs) and programmed cell death 1 ligand 1 (PD-L1) are established prognostic and predictive biomarkers for certain breast cancer subsets. However, their association with the immune response complexity is not fully understood. Therefore, we analyzed the association between the immune cell fractions in breast cancer tissues and histologically assessed TIL (hTIL) and PD-L1 (hPD-L1). Forty-five tumor and eighteen blood samples were collected from patients with breast cancer. Total leukocyte counts, frequency of 11 immune cell populations, and PD-L1 expression in each cell fraction were evaluated by flow cytometry. TILs and PD-L1 were assessed by hematoxylin and eosin staining and immunohistochemistry, respectively. A higher hTIL score showed association with increased leukocyte infiltration, higher CD4^+^ and CD8^+^ T cell proportions, and lower natural killer and natural killer T cell proportions. PD-L1 was highly expressed in nonclassical monocytes, monocyte/macrophages, myeloid-derived suppressor cells, myeloid dendritic cells, dendritic cells, and other lineages in tumors. hPD-L1 positivity reflected PD-L1 expression accurately in these fractions, as well as increased leukocyte infiltration in tumors. These results indicate that hTILs reflect differences in the immune responses in the tumor microenvironment, and certain immune cell fractions are favorably expressed in the PD-L1 pathway in breast cancer microenvironments.

## Introduction

Breast cancer is the most common malignancy among women worldwide. Although progress has been made regarding multimodal treatment comprising surgery, systemic therapy, and radiation therapy, the cure of advanced and recurrent diseases continues to be difficult^1^. The recent success in the clinical use of immune checkpoint inhibitors for multiple cancers has attracted attention in tumor immunology, and an improved understanding of tumor immunology may inform the development of new treatment strategies or effective use of existing therapies^2^.

Histologically assessed tumor-infiltrating lymphocytes (hTILs) can provide prognostic information for diverse solid tumor types and may be of value in predicting response to treatment^3^. In breast cancer, amid some controversy, hTIL has been detected more frequently in the triple-negative subtype or human epidermal growth factor receptor-2 (HER-2)-positive subtype than in the luminal subtype^3^ and is correlated with clinicopathological factors other than subtypes^4,5^. hTIL is associated with the prognosis of disease-free and overall survival in triple-negative and HER-2-positive subtypes^4,6^. Additionally, hTIL has been proposed as a predictor of response to neoadjuvant chemotherapy in all molecular subtypes^7^. Programmed cell death 1 ligand 1 (PD-L1) is expressed in immune cells, including T cells, B cells, macrophages, monocytes, and dendritic cells, as well as in tumor cells. PD-L1 can bind to the programmed cell death protein 1 (PD-1) expressed on activated T cells. Interactions with PD-L1 enable PD-1 signaling to counter the activation of T cells during the effector phase of the immune response^8,9^. Histologically assessed PD-L1 (hPD-L1) is expressed in HER-2 and triple-negative subtypes more frequently than in luminal subtypes and is correlated with poor prognoses, high histological grades, and lymphatic vessel invasions^10^. hPD-L1 has been established clinically as a predictor of atezolizumab efficacy in triple-negative advanced breast cancer^11^. Recently, different types of immune cell subsets evaluated primarily via immunohistochemistry (IHC) have been found to be associated with various clinicopathological factors or prognoses. These evaluations indicate their clinical significance in breast cancer^12–28^ and are summarized in Supplementary Table S1.

Therefore, hTIL and hPD-L1 have been established as biomarkers for breast cancer; recent studies have shifted their attention to the various immune cell subsets that make up hTILs. However, the complexity of the multiple types of immune cells in TIL or PD-L1-expressing cells is not yet fully understood because of the technical difficulties in detecting multiple types of immune cells in the tissues using conventional IHC. Therefore, we first used multicolor flow cytometry (FCM) to assess the multiple immune cell fractions in breast cancer tissue and blood. We then analyzed the association between hTIL and hPD-L1. By performing a systematic analysis of the immune cell composition, we aimed to show that specific immunological profiles of the breast cancer microenvironment were represented histologically by hTIL and hPD-L1.

## Methods

### Patients

Forty-seven tumor samples from the primary site and 19 matched blood samples were obtained from patients with breast cancer regardless of clinicopathological factors or treatment histories, except for patients with distant metastases or complete clinical responses to neoadjuvant chemotherapy. No patient in this study received irradiation or endocrine therapy before surgery. Following an amendment of the study protocol in July 2016, blood sample collection commenced; therefore, there were cases with no blood samples. Clinicopathological data, including menopausal statuses, histories of preoperative chemotherapy, histological types, invasive tumor sizes, lymph node statuses, lymphatic involvement, vascular involvement, histological grades, estrogen receptor (ER) statuses, progesterone receptor (PgR) statuses, HER-2 statuses, and Ki67 labeling indexes, were collected by reviewing case records. Histological grades were evaluated according to the method described by Robbins et al.^29^. ER, PgR, and HER-2 statuses were evaluated using IHC staining. The cutoff value for ER and PgR positivity was set at ≥ 10%^30^. The HER-2 status was determined according to the ASCO CAP guideline 2013^31^. For reasons mentioned later, 45 tumor samples and 18 blood samples were included in the analysis. The clinicopathological characteristics of the entire cohort and the cohort with blood samples are presented in Supplementary Tables S2 and S3, respectively.

### Tumor-infiltrating lymphocyte/peripheral blood mononuclear cell (PBMC) preparation

Tumor and blood samples were collected simultaneously during surgery. Tumor samples were mechanically dissociated on ice with 10% fetal bovine serum-phosphate-buffered saline (FBS-PBS), filtered using a 70-micron strainer, and washed with 10% FBS-PBS. All blood samples were collected using a collection tube containing ethylenediaminetetraacetic acid-2Na. For both the tumor and blood samples, mononuclear cell components were separated using density-gradient centrifugation with a Ficoll-Paque PLUS (Cytiva Inc., Tokyo, Japan) according to the manufacturer’s instructions. Then, they were suspended in a CELLBANKER I (Takara Bio Inc. Shiga, Japan) and stored in liquid nitrogen.

### Flow cytometry (FCM) analysis

The cryopreserved TILs and PBMCs were thawed and washed with 10% FBS-PBS. The cell suspensions were then processed for surface staining with an antibody cocktail (Supplementary Table S4) for 20 min at 4 °C. The cells were washed with PBS containing 2% FBS and resuspended in CellFix (BD Biosciences, Franklin Lakes, NJ, USA). The stained cells were detected using an LSR II Fortessa with FACS Diva software (BD Biosciences). All analyses were performed using FlowJo software (BD Biosciences). The gating strategy is shown in Supplementary Fig. S1. Immune cell fractions were classified into the following according to the definitions shown in Supplementary Table S5: leukocytes, total T cells (Total T), CD4^+^ T cells (CD4^+^ T), CD8^+^ T cells (CD8^+^T), B cells (B), monocytes/macrophages (Mo/Mφ), nonclassical monocytes (CD16^+^ Mo), myeloid-derived suppressor cells (MDSCs), dendritic cells (DCs), myeloid dendritic cells (mDCs), natural killer (NK), minor NK cells, and natural killer T cells (NKT). Two authors (TH and MY) verified the raw data independently to exclude input errors. Two cases with a low number of living cells (count < 1000) in the FCM analysis of the tumor tissue were excluded. A blood sample that was identical to one of the two cases was also excluded. The leukocyte density, based on the weight of the tissue fragment and number of viable CD45^+^ cells, was determined using a previously described method^32^. For the immune cell fraction, we determined both the percentages of each fraction in the leukocytes (% in leukocytes) and densities based on the weights of the tissue fragments (count/g).

### Histological evaluation of tumor immunity-related biomarkers

We evaluated the histological tumor immunity-related biomarkers, as described previously^33–36^. Briefly, the percentages of stromal TILs were evaluated using 4-µm sections from formalin-fixed specimens stained with hematoxylin and eosin using a light microscope at × 200–400 magnification. Stromal TILs were defined as mononuclear cells localized in the stromal tissue of breast cancer. The stromal TIL count was categorized, according to the International TILs Working Group guideline, into three grades: low (0–10%), intermediate (10–40%), and high (40–90%), and scored from 0 to 2. The denominator used to determine the TIL grade was the stromal tissue area. PD-L1 expression was assessed using IHC with rabbit monoclonal anti-PD-L1 clone SP142 (prediluted; Spring Bioscience, Pleasanton, CA, USA) and the Ventana Benchmark ULTRA auto staining machine. Tumors with ≥ 1% immune cells with cytoplasmic and/or membrane PD-L1 staining were determined to be PD-L1 positive. Stromal TIL counts and PD-L1 expression were evaluated by two evaluators (CH and SK). The histologically assessed TIL and PD-L1 were described as hTIL and hPD-L1, respectively, to distinguish them from the FCM-assessed data. In one case, the hTIL and hPD-L1 could not be evaluated because the remaining tumor tissue was insufficient. Therefore, hTIL and hPD-L1 were analyzed in the remaining 44 patients.

### Statistical analyses

All statistical analyses and graph drawings were performed using GraphPad Prism ver. 9.1.0 software. The normality of the FCM data was tested using the D'Agostino–Pearson normality test. The correlation analyses between groups were performed using Spearman’s rank correlation coefficient. For the comparison of the paired samples of the tumor and blood, the Wilcoxon test was used. For the comparison of the two unpaired groups, the Mann–Whitney *U* test was used. A Chi-square test was used to compare clinicopathological factors of the hTILs and hPD-L1. A Fisher’s exact test was used when the Chi-square test indicated a significant *P*-value (*P* < 0.05) and there were cells with a sample size of ≤ 5.

### Ethical approval and participant consent

This study was conducted at Tokai University Hospital and approved by the Ethics Committee, which conforms to the provisions of the Declaration of Helsinki (Accepted project No. 16R-279). The patients were enrolled from May 2015 to April 2019, and written informed consent was obtained from all patients.

## Results

### Distribution of leukocyte and immune cell fraction determined by FCM

**Figure 1:**
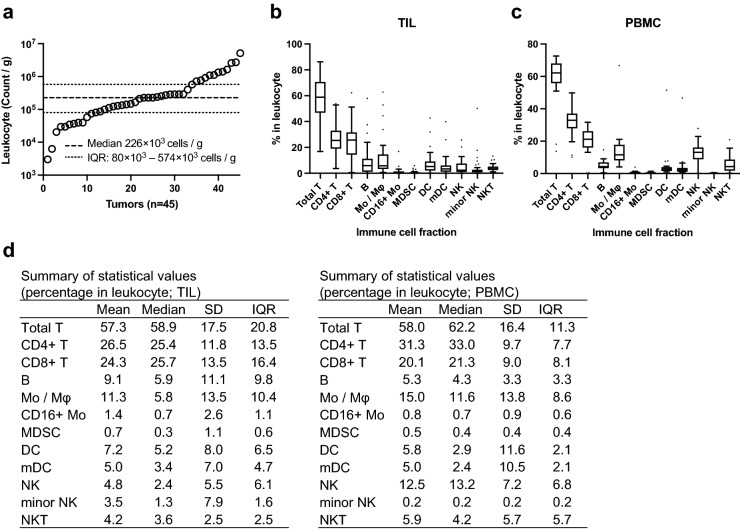
Summary of leukocytes and immune cell fractions distribution determined using flow cytometry (FCM). Tumor and blood samples were assessed using multicolor FCM, and leukocyte and 11 types of immune cell fractions in the samples were analyzed. (**a**) Distribution of leukocyte density (count/g) determined by FCM in 45 breast tumors. The graph shows the medians, 25th percentile, and 75th percentile. (**b**, **c**) Percentages of each immune cell fraction in tumors (n = 45) and blood samples (n = 18) showed as Tukey box plots. (**d**) Statistical values are summarized for the percentages of each immune cell fraction in the tumor tissues (n = 45).

Leukocyte densities based on the weights of the tissue fragments and the number of viable CD45^+^ cells were determined in the whole cohort (n = 45). The mean density and interquartile range of the tumor-infiltrating leukocytes were 226 × 10^3^ cells/g and 80 × 10^3^ to 574 × 10^3^ cells/g, respectively (Fig. 1a). For the immune cell fraction in the leukocytes of the tumor tissues (TILs), the main population comprised CD8^+^ T, CD4^+^ T, Mo/Mφ, and B cells (Fig. 1b, d). A similar trend was observed in the leukocyte composition in the blood (PBMC), showing that the main population comprised CD8^+^ T and CD4^+^ T cells (Fig. 1c, d).

### Histologically assessed TIL is associated with the degree of leukocyte infiltration in tumor tissue and leukocyte composition

**Figure 2:**
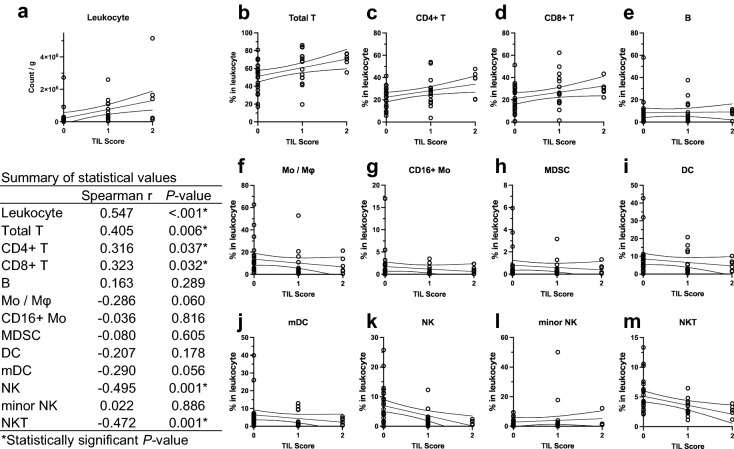
hTIL is associated with the degree of leukocyte infiltration in tumor tissues and leukocyte composition. For cases with tumor tissue samples, a correlation analysis was performed for (**a**) hTIL scores and leukocyte densities (count/g) in tumor tissues, (**b**–**m**) hTIL scores, and percentages of each immune cell fraction in the tumor tissues. The X- and Y-axes show the hTIL scores and (**a**) leukocyte densities (count/g) or (**b**–**m**) the percentages of the immune cell fractions in the tumor tissue, respectively. The lines in the graph indicate the regression line with a 95% confidence band. The relationship between these values was analyzed using the Spearman correlation test. Values of *P* < 0.05 are considered statistically significant. Statistical values are summarized in the table on the left side of the figure.

The assessment of clinicopathological characteristics using the hTIL score showed that higher hTIL scores were associated with high-grade tumors, ER negativity, higher Ki67-positive ratios, and hPD-L1 positivity (Table 1). For cases with tumor tissue samples, correlation analysis was performed for the hTIL scores and leukocyte densities (count/g) in the tumor tissues, which were strongly positively correlated with each other (Fig. 2a). Furthermore, the hTIL scores showed a positive correlation with the densities (count/g) of all immune cell fractions in the tumor tissues, except for NK (Supplementary Fig. S2). Correlation analysis was also performed for the hTIL scores and percentages of each immune cell fraction in the tumor tissues. We observed positive correlations between the hTIL scores and percentages of total T, CD4^+^ T, and CD8^+^ T (Fig. 2b–d), but negative correlations between the hTIL scores and NK and NKT (Fig. 2k, m), showing that hTIL was associated with the degree of leukocyte infiltration in tumor tissue and leukocyte composition. There were no correlations between the hTIL scores and the percentages of B, Mo/Mφ, CD16 + Mo, MDSC, DC, mDC, and minor NK cells (Fig. 2e–j, l).

**Table 1:** Clinico-pathological characteristics by hTIL score.

	hTIL score 0 (N = 23)	hTIL score 1, 2 (N = 21)	*p-*value		hTIL score 0 (N = 23)	hTIL score 1, 2 (N = 21)	*p-*value
**Menopausal status**			0.512	**Vascular invasion**			0.089
Unknown	0	1		Unknown	1	0	
Post	16 (69.6%)	12 (60.0%)		Negative	17 (77.3%)	20 (95.2%)	
Pre	7 (30.4%)	8 (40.0%)		Positive	5 (22.7%)	1 (4.8%)	
**Neo-adjuvant therapy**			0.155	**Histological grade**			0.011*
Absent	17 (73.9%)	19 (90.5%)		Unknown	2	3	
Present	6 (26.1%)	2 (9.5%)		Grade 3	2 (9.5%)	9 (50.0%)	
**Histological type**			0.544	Grade 1, 2	19 (90.5%)	9 (50.0%)	
IDC	20 (87.0%)	18 (85.7%)		**ER status**			0.002*
ILC	1 (4.3%)	0 (0.0%)		Positive	14 (60.9%)	3 (14.3%)	
Special	2 (8.7%)	3 (14.3%)		Negative	9 (39.1%)	18 (85.7%)	
**Invasive tumor size**			0.631	**PgR status**			0.269
Unknown	1	0		Positive	5 (21.7%)	2 (9.5%)	
≥ 20 mm	19 (86.4%)	17 (81.0%)		Negative	18 (78.3%)	19 (90.5%)	
< 20 mm	3 (13.6%)	4 (19.0%)		**HER2 status**			0.062
**Lymph node metastasis**			0.443	Unknown	0	1	
Unknown	1	0		Positive	6 (26.1%)	1 (5.0%)	
Negative	10 (45.5%)	12 (57.1%)		Negative	17 (73.9%)	19 (95.0%)	
Positive	12 (54.5%)	9 (42.9%)		**Ki67**			0.036*
**Lymphatic invasion**			0.897	≥ 20%	14 (60.9%)	19 (90.5%)	
Unknown	1	0		< 20%	9 (39.1%)	2 (9.5%)	
Negative	9 (40.9%)	9 (42.9%)		**PD-L1**			< .001*
Positive	13 (59.1%)	12 (57.1%)		Unknown	0	0	
				Positive	7 (30.4%)	18 (85.7%)	
				Negative	16 (69.6%)	3 (14.3%)	

*Statistically significant *p*-value.

### Histologically assessed PD-L1 expression is associated with leukocyte infiltration in tumor tissue and reflects PD-L1 expression in certain immune cell fractions

**Figure 3:**
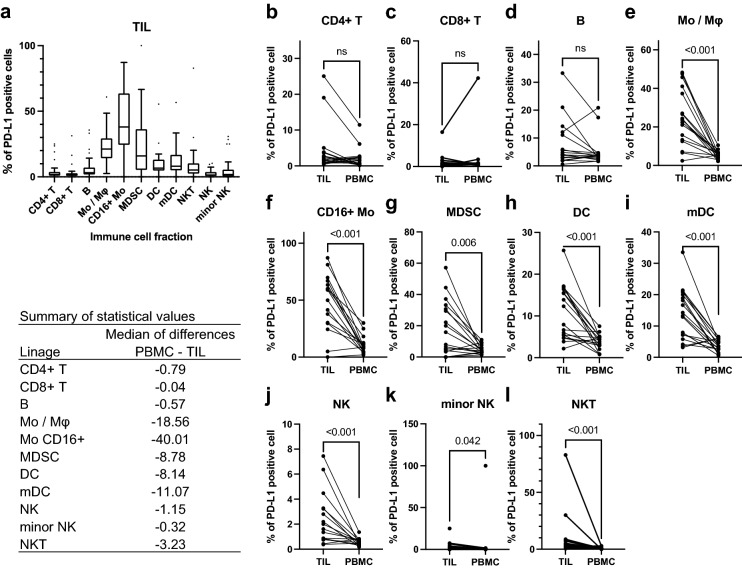
PD-L1 is preferentially expressed in part of the immune cell fraction, and its expression is higher in tumor-infiltrating leukocytes than in blood. The percentages of PD-L1-positive cells of each immune cell fraction were determined in tumor tissues (TIL) and blood (PBMC). (**a**) The percentages of PD-L1-positive cells in each immune cell fraction present in tumor tissue are shown in Tukey box plots. (**b**–**l**) For the cases with matched samples of blood and tumor tissue, the percentages of PD-L1-positive cells of each immune cell fraction were compared in tumor tissue (TIL) and blood (PBMC) using the Wilcoxon test. Values of *P* < 0.05 are considered statistically significant. The actual *P*-values are shown in the graphs. The medians of the differences are summarized in the table on the left side of the figure.

**Figure 4:**
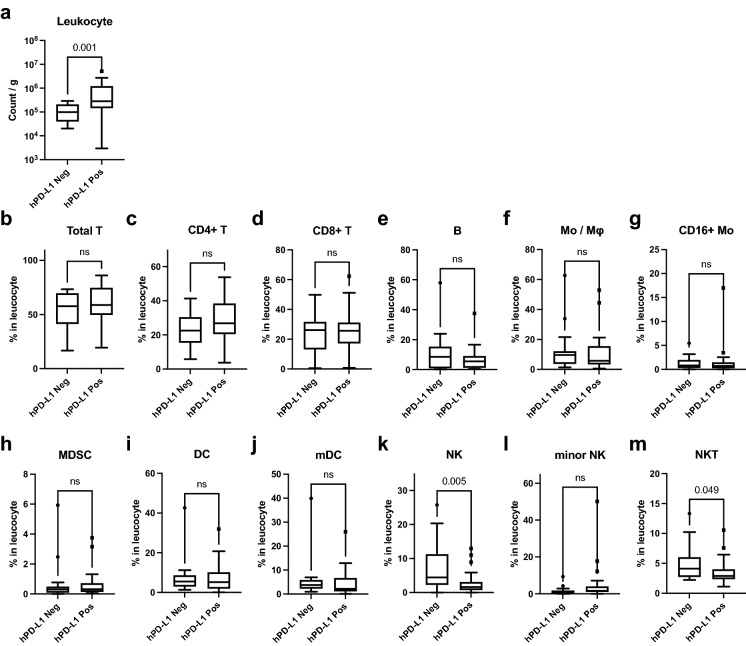
hPD-L1 positivity is associated with increased leukocyte infiltrations in the tumor tissue and partly with the immune cell composition. For cases with tumor tissue samples, (**a**) leukocyte densities (count/g) and (**b**–**m**) the percentages of the immune cell fractions in the tumor tissues were compared between the hPD-L1-negative cases and hPD-L1-positive cases using the Mann–Whitney *U* test. The data are presented using Tukey box plots. Values of *P* < 0.05 were considered statistically significant. The actual *P*-values are shown in the graphs.

**Figure 5:**
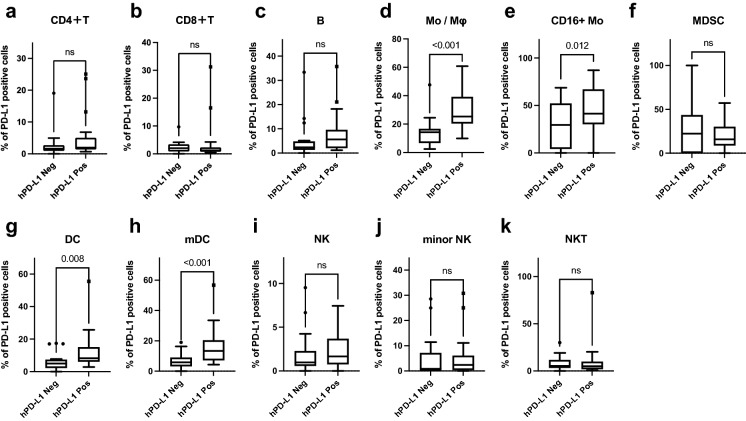
hPD-L1 positivity is associated with percentages of the PD-L1 positive cells in certain immune cell fractions. (**a**–**k**) For cases with tumor tissue samples, the percentages of PD-L1-positive cells in each immune cell fraction of the tumor tissues in the hPD-L1-negative and hPD-L1-positive cases were compared using the Mann–Whitney *U* test. The data are presented using Tukey box plots. Values of *P* < 0.05 are considered statistically significant. The actual *P*-values are presented in the graphs.

The percentages of the PD-L1-positive cells in each immune cell fraction were determined for the tumor tissues (TILs) and blood (PBMC) using FCM. The percentages of the PD-L1-positive cells were high in CD16^+^ Mo, Mo/Mφ, MDSC, mDC, DC populations (Fig. 3a). For cases with matched samples of blood and tumor tissue, the percentages of the PD-L1-positive cells in each immune cell fraction of the tumor tissues and blood were compared. The percentages of the PD-L1 positive cells were significantly higher in tumor tissues than in blood for all lineages except for the lymphoid fractions (Fig. 3b–l). Next, we investigated the relationship between hPD-L1 and the immunological profiles of the tumor tissues. Comparisons between the leukocyte densities (count/g) in the tumor tissues of the hPD-L1-negative and -positive cases showed that hPD-L1 positivity was associated with increased leukocyte infiltrations in tumor tissues (Fig. 4a). Similarly, hPD-L1 showed a positive correlation with the densities (count/g) of all immune cell fractions in the tumor tissues, except for B and NK cell fractions (Supplementary Fig. S3). With regard to the immune cell composition in the tumor tissues, although hPD-L1 positivity was associated with a lower percentage of NK and NKT, it was not correlated with the percentages of other lineages (Fig. 4b–m). For tumor tissue samples, percentages of the PD-L1 positive cells in each immune cell fraction in the hPD-L1-positive and -negative cases were compared. We found that hPD-L1 positivity showed a positive association with the percentages of the PD-L1 positive cells in some of the immune cell fractions, including Mo/Mφ, CD16^+^ Mo, DC, and mDC, but not with the other lineages (Fig. 5a–k). These data suggest that hPD-L1 expression reflects leukocyte infiltration in the tumor tissues and PD-L1 expression in certain immune cell fractions.

## Discussion

In this study, we evaluated multiple immune cell fractions in both breast cancer tissues and matched blood samples. In the comprehensive analysis of the association between each immune cell fraction with hTIL and hPD-L1, we demonstrated for the first time that these biomarkers reflect not only the degree of immune cell infiltration in the tumor but also the proportion of a particular immune cell subset. We also showed that hPD-L1 reflects PD-L1 expression in certain immune cell fractions, including Mo/Mφ, CD16^+^ Mo, DC, and mDC.

To the best of our knowledge, most of the analyses of the immune cell compositions of breast cancer tissues using a multicolor FCM had 10 colors or less^32,37^, and there were only two studies with more than 11 colors^38,39^. Although the reactivities of the labeled antibodies were not always the same, and a direct comparison was not possible, a similar distribution of the leukocyte infiltrations was observed in the tumor tissue in our study and a previous study with a distribution median of 218 CD45^+^ TIL/mg of tumor tissue (interquartile range: 85–445 CD45^+^ TIL/mg)^32^. Although there are very few reports of systematic examinations of leukocyte compositions in breast cancer tissue, studies have reported the ratio of total T to be 86% (mean)^32^ or 75% (median)^38^ of the leukocytes (CD45^+^ cells) in breast cancer tissues, suggesting that T cells account for the majority of TILs^40^. In our study, the proportion of total T cells in the leukocytes in the tumors was 57.3% (mean), which was slightly lower than that in previous reports, probably owing to the difference in the antibody used and gating strategy. In three previous studies, the proportions of CD19^+^ B cells in CD45^+^ TIL were found to be 8% (mean), 4.58% (median), and approximately 10% (mean), respectively^32,37,38^, which were similar to our results. With regard to other lineages, the findings of a previous study showed that the proportions of CD14^+^/CD40^+^/CD163^+^ M2 macrophages were 0.06% (median), those of CD11b^+^/CD15^+^/HLA-DR-MDSCs were 1.19% (median), and those of CD56^+^ NK were 2.33% (median)^38^. However, there were only a few studies, and the definitions of each lineage did not match those in our study; therefore, valid comparisons could not be made. No comparable reports were found for the remaining lineages.

For cases with matched samples of blood and tumor tissue, we exploratorily analyzed the association between the immune cell composition of blood and breast cancer tissues. Immune composition of the blood partially correlated with that of tumor tissues (Supplementary Fig. S4a–l), and the percentages of the immune cell fractions in tumor tissues and blood presented certain differences (Supplementary Fig. S5a–l). However, phenotypes, such as memory and naïve T cells, which have a different composition in blood and tissue, were not analyzed in this study. Therefore, the relationship between immune cell composition in blood and breast cancer tissue cannot be thoroughly discussed with our current data, and the implications of these results will be considered in future studies. Although we have investigated the relationship between blood immune cell composition and hTIL and hPD-L1, no significant associations were observed (Supplementary Fig. S6 and S7). Similarly, no significant associations were identified between hPD-L1 and FCM-assessed PD-L1 positive ratios in the immune cell fraction of blood (Supplementary Fig. S8).

Although IHC has been used for histological evaluation of TIL in previous studies, the target antigens or clones of antibodies used in these studies are diverse, as it is not standardized nor routinary in clinical settings^3,36^. In addition, TIL evaluation by hematoxylin and eosin staining has been recommended by the International TILs Working Group to assess tumor-immunological status in clinical practice. However, the kind of immunological tumor status reflected by this simple index (hTIL) has not been fully investigated yet. Thus, one of the primary purposes of this study was to investigate the significance of hTIL assessed according to the mentioned guideline. Although there was a significant difference between the subtypes, hTIL was correlated with certain clinicopathological factors, including subtypes^3–5^, prognoses, and responses to chemotherapy^4,6,7^, in breast cancer. Numerous studies have also reported that ER-positive breast cancer is the least immune-infiltrated subtype, which is consistent with our results^2,5^. However, there are certain controversies regarding other clinicopathological factors, and results vary among different studies^2,4,5,41^. No studies have systematically assessed the relationship between hTIL and the immune cell fraction using FCM. In our study, hTIL assessed following the guidelines of the International TILs Working Group showed a positive correlation with the infiltration (count/g) of all immune cell fractions in tumor tissue except for NK (Supplementary Fig. S2). Moreover, cases with a higher hTIL score have a higher percentage of the lymphocyte fraction but a lower fraction of NK and NKT (Fig. 2). These data suggest that when TIL increases in tumor tissue, lymphocytic fraction shows a prominent increase, and the percentages of cells with a relatively small increase such as NK and NKT are decreased.

As mentioned previously, PD-L1 plays a significant role in immune tolerance mechanisms^8,9^, and its expression is suggested to reflect ongoing (or active) immune responses in addition to immunosuppression via the PD-1/PD-L1 pathway^39^. hPD-L1 was shown to correlate with certain clinicopathological factors, including subtypes^10^. It is also a clinically approved predictive marker for atezolizumab in triple-negative advanced breast cancer^11^. In the present study, hPD-L1 positivity was associated with ER negativity and relatively high hTIL scores but no other factors, probably owing to the small cohort size (Supplementary Table S6). Although PD-L1 expression in multiple types of immune cells or tumor cells has been reported^8,9^, there is no consensus as to which immune cell fraction is responsible for the substantial function of the PD-L1 pathway in breast cancer. The findings of only one report that evaluated PD-L1 expression in CD4 ^+^ T, CD8 ^+^ T, and B cells showed that the overall proportion of the PD-L1-positive TILs was very low and could only be detected in a small number of tumors^39^. In the present study, we found that PD-L1 was preferentially expressed in CD16 + Mo, Mo/Mφ, MDSC, mDC, DC, and the percentages of the PD-L1 positive cells of these lineages were significantly higher in tumor tissues than in blood, suggesting that these fractions are involved primarily in the PD-L1 pathway in breast cancer tissue. Additionally, we found that hPD-L1-positive tumors exhibited increased leukocyte infiltration in tumor tissues, and hPD-L1 reflected PD-L1 expression in Mo/Mφ, CD16^+^ Mo, DC, and mDCs. These results suggested that hPD-L1 expression can indicate the activation status of the immune tolerance mechanism that occurs in certain immune cell fractions such as CD16^+^ Mo, Mo/Mφ, MDSC, mDC, and DC in response to increased immune cell infiltration, mainly effector cells that secrete interferon-gamma to induce PD-L1 expression on various cells, into the breast cancer microenvironment. The significance of hPD-L1 expression in tumor cells has been previously reported^9,33,42^. In this study, the hPD-L1 expression in immune cells was determined using PD-L1 antibody clone SP142, following the clinically accepted diagnostic criteria^11^. Accordingly, tumors with 1% or more immune cells with PD-L1 staining were determined to be hPD-L1 positive, which was identified only in 2 out of 44 cases (4.5%). Although this frequency agrees with that reported in previous reports^33^, the small case number makes it difficult to perform a meaningful statistical analysis. Therefore, to gain insights into the significance of PD-L1 expression in tumor cells, future studies involving a large number of cases should be conducted.

This study has several limitations. The number of patients enrolled was relatively small. A pilot study empirically found that the number of cells required for FCM was not sufficient in cases of ER-positive breast cancer, especially in cases with lower Ki67s. Cases of small tumor sizes and post-NAC with complete pathological responses were excluded owing to technical problems in collecting the tumor tissues. Therefore, there was an inevitable bias in the enrollment of the cases; it differed from the general breast cancer cohort in terms of larger invasive tumor sizes, more ER-negative cases, and higher Ki67 cases (Supplementary Table S2). Although, as mentioned above, the significance of the TIL is suggested to vary between subtypes, a subgroup analysis could not be performed because of the small sample size. In future studies, the inclusion of more samples and more detailed analyses are recommended. Furthermore, the FCM data contained outliers; however, we could not compare the values because of a lack of suitable reports; hence, the biological reliability of the outliers cannot be ruled out. Therefore, all analyses were performed without the omission of outliers. However, to ensure the reliability of our analyses, we identified the outliers using the ROUT method, excluded them, and reperformed all statistical analyses. The results did not differ much (Supplementary Tables S7–S17) compared with those obtained by including the outliers, indicating the reliability of the results.

## Conclusions

A comprehensive analysis of the immune cell fractions revealed the immunological profiles of breast cancer tissue represented by hTIL or hPD-L1. Our findings indicated that hTIL reflected the amount of immune cell infiltration, as well as the proportion of a particular immune cell subset. Immune cell fractions, such as CD16 ^+^ Mo, Mo/Mφ, MDSC, mDC, DC, were preferentially involved in the PD-L1 pathway in breast cancer microenvironments. Additionally, we demonstrate that hPD-L1 represented the PD-L1 expression in these immune cell fractions. Collectively, our findings provide a basic understanding of the immune response in the breast cancer microenvironment and contribute to the development of tumor immunology.

## Supplementary Information

Supplementary Information.

## Data Availability

The datasets generated and/or analyzed during the current study are available upon reasonable request to the corresponding author.
