# Supplementary material for: Immunological profiles of the breast cancer microenvironment represented by tumor-infiltrating lymphocytes and PD-L1 expression
Source: Sci Rep. 2022 May 16;12:8098. doi: 10.1038/s41598-022-11578-x (PMC9110375; doi:10.1038/s41598-022-11578-x)
Supplement: Supplementary file 1 — Supplementary Information. [file 41598_2022_11578_MOESM1_ESM.docx]

**Immunological profiles of the breast cancer microenvironment represented by tumor-infiltrating lymphocytes and PD-L1 expression**

Toru Hanamura, Shigehisa Kitano, Hiroshi Kagamu, Makiko Yamashita, Mayako Terao, Banri Tsuda, Takuho Okamura, Nobue Kumaki, Katsuto Hozumi, Naoki Harada, Takayuki Iwamoto, Chikako Honda, Sasagu Kurozumi, Naoki Niikura^*^

**Supplementary information**


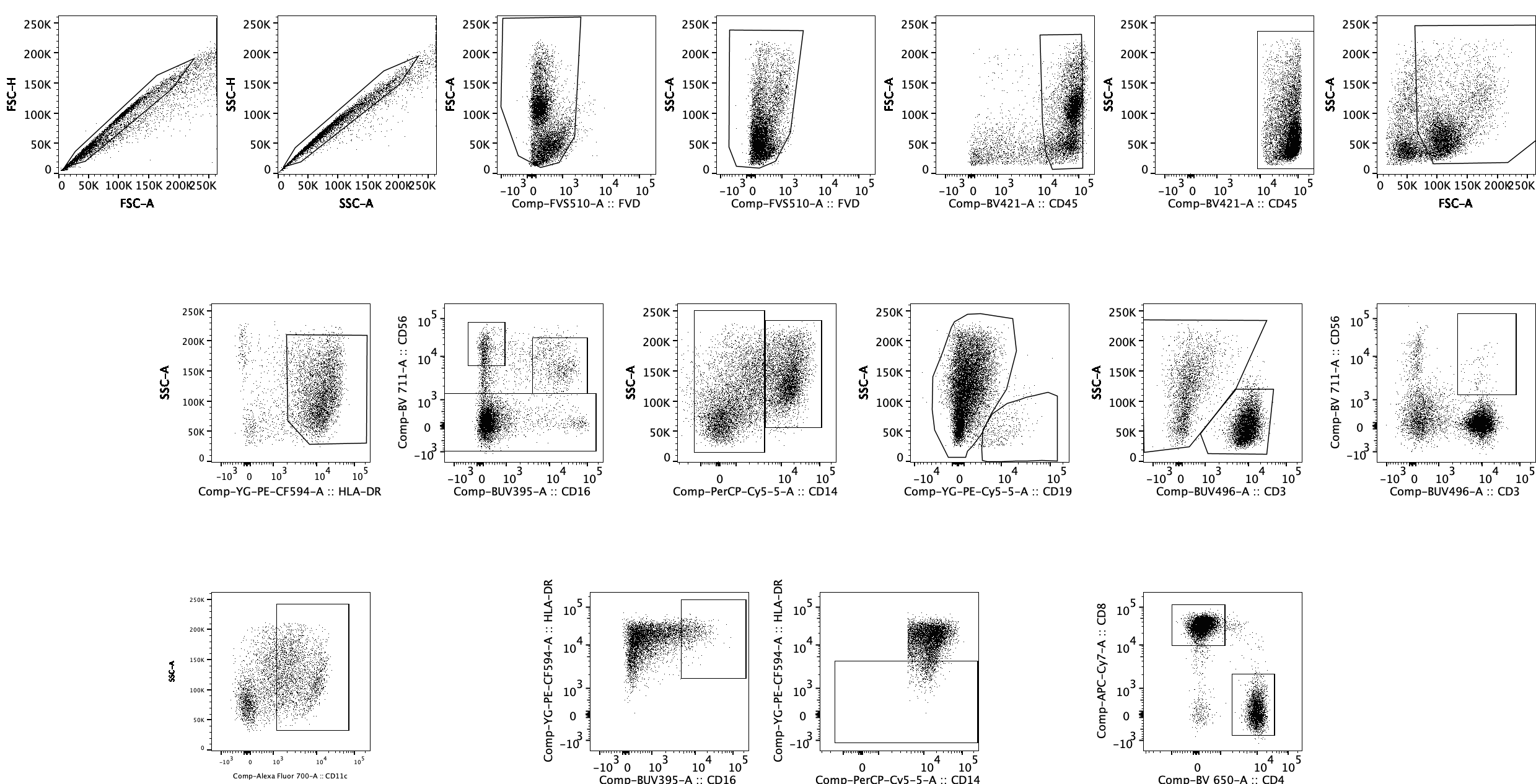


Leukocyte

NKT

total T

B cell

Mo / Mφ

CD16^+^ Mo

MDSC

CD4^+^ T

minor NK

NK

mDC

FVD

FVD

CD45-BV421

CD45-BV421

FSC-A

CD3-BV421

CD56-BV711

CD56-BV711

CD3-BV421

SSC-A

SSC-A

CD19-PE/Cy5.5

SSC-A

CD14-PerCP/Cy5.5

CD16-BUV395

HLA-DR-PE/CF594

SSC-A

CD8^+^ T

CD11c-Alexa Fluor 700

SSC-A

HLA-DR-ECD

CD16-BUV395

CD14-PerCP/Cy5.5

HLA-DR-ECD

CD4-BV650

CD8-APC/Cy7


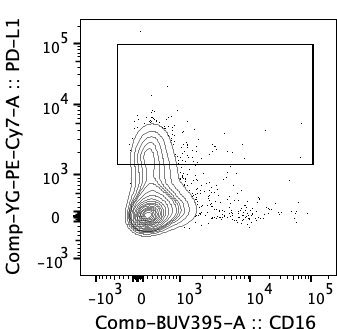


PD-L1^+^ mDC

PD-L1-PE/Cy7

CD16-BUV395

DC

**Supplementary Figure S1. Lymphocyte gating strategy for the identification of each lineage and PD-L1-positive cells.** Appropriate isotype controls served as the cutoff between positive and negative cells. A positive gate was set to include less than 0.5% of cells in each specimen with a matched isotype control. Each population was defined as CD4^+^ T cell, CD3^+^CD4^+^; CD8^+^ T cell, CD3^+^CD8^+^; B, CD3^-^CD19^+^; Monocyte/Macrophage, CD3^-^CD19^-^CD14^+^; CD16^+^ Mo, CD3^-^CD19^-^CD14^+^CD16^+^; MDSC, CD3^-^CD19^-^ CD14^+^HLADR^low^; NK, CD3^-^CD19^-^CD14^-^CD56^low^CD16^+^; minor NK, CD3^-^CD19^-^CD14^-^CD56^-^CD16^high^; mDC, CD3^-^CD19^-^CD14^-^CD56^-^HLADR^+^CD11c.

**
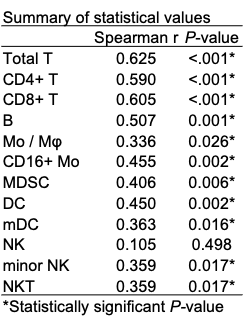
**

**a**

**b**

**c**

**d**

**e**

**f**

**g**

**h**

**i**

**j**

**k**

**l**

**Supplementary Figure S2. Correlation of histologically assessed tumor-infiltrating lymphocytes (hTILs) with infiltration of immune cell fractions in tumor tissue.** (a–l) For cases with tumor tissue samples, a correlation analysis was performed for the hTIL scores and densities (count/g) of each immune cell fraction in the tumor tissues. The X- and Y-axes show the hTIL scores and densities (count/g) of each immune cell fraction. The lines in the graph indicate the regression line with a 95% confidence band. The relationship between values was analyzed using a Spearman correlation test. Values of *P* < 0.05 were considered statistically significant. The statistical values are summarized in the table on the left side of the figure.

**a**

**b**

**c**

**d**

**e**

**f**

**g**

**h**

**i**

**j**

**k**

**l**

**Supplementary Figure S3. Correlation of histologically assessed programmed cell death 1 ligand 1 (hPD-L1) with infiltration of immune cell fractions in tumor tissues.** (a–l) For cases with tumor tissue samples, the densities (count/g) of each immune cell fraction in the tumor tissues were compared ​between the hPD-L1-negative cases and hPD-L1 positive cases using a Mann–Whitney *U* test. The data are shown using Tukey box plots. Values of *P* < 0.05 were considered statistically significant. The actual *P*-values are shown in the graphs.


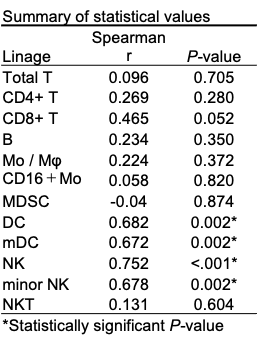

**a**

**b**

**c**

**d**

**e**

**f**

**g**

**h**

**i**

**j**

**k**

**l**

**Supplementary Figure S4. Correlation between immune cell composition of blood and tumor tissue.** (a–l) For cases with matched samples of blood and tumor tissues, a correlation analysis was performed for the percentage of each immune cell fraction. The X- and Y-axes show the percentage of immune cell fractions for the tumor tissue (TIL) and blood (PBMC), respectively. The lines in the graph indicate the regression line with a 95% confidence band. The relationship between these values was analyzed using the Spearman correlation test. Values of *P* < 0.05 were considered statistically significant. Statistical values are summarized in the table on the left side of the figure.


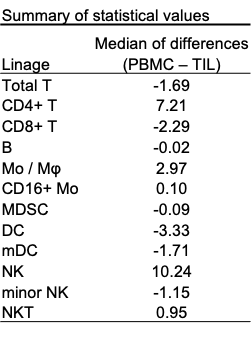

**a**

**b**

**c**

**d**

**e**

**f**

**g**

**h**

**i**

**j**

**k**

**l**

**Supplementary Figure S5. Percentage of immune cell fractions in tumor tissue and blood.** (a–l) For 18 cases with matched samples of blood and tumor tissues, the percentages of each immune cell fraction of tumor tissues (TIL) and blood (PBMC) were compared using the Wilcoxon test. Values of *P* < 0.05 were considered to be statistically significant. The actual *P*-values are shown in the graphs. The medians of the differences are summarized in the table on the left side of the figure.

**
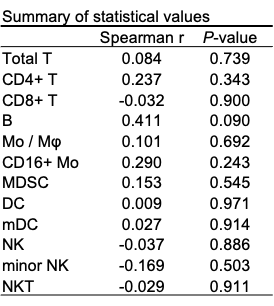
**

**a**

**b**

**c**

**d**

**e**

**f**

**g**

**h**

**i**

**j**

**k**

**l**

**Supplementary Figure S6. Association between the hTIL and immune cell composition of blood.** (a–l) For cases with a blood sample, a correlation analysis was performed for the hTIL scores and percentages of each immune cell fraction in blood. The X- and Y-axis show the hTIL scores and percentages of the immune cell fractions. The lines in the graph indicate the regression line with a 95% confidence band. The relationship between these values was analyzed using a Spearman correlation test. Values of *P* < 0.05 were considered statistically significant. The statistical values are summarized in the table on the left side of the figure.

**a**

**b**

**c**

**d**

**e**

**f**

**g**

**h**

**i**

**j**

**k**

**l**

**Supplementary Figure S7. Association between hPD-L1 and immune cell composition of blood.** (a–l) For cases with blood samples, the percentages of each immune cell fraction in blood were compared between the hPD-L1-negative cases and hPD-L1 positive cases using the Mann–Whitney *U* test. The data are shown using Tukey box plots. Values of *P* < 0.05 were considered statistically significant. The actual *P*-values are shown in the graphs.

**a**

**g**

**b**

**c**

**d**

**e**

**f**

**h**

**i**

**j**

**k**

**Supplementary Figure S8. Association between hPD-L1 and the percentage of PD-L1-positive cells in the immune cell fraction of blood.** (a–k) For cases with blood samples, the percentages of PD-L1 positive cells in each immune cell fraction in blood were compared between the hPD-L1-negative cases and hPD-L1-positive cases using the Mann–Whitney *U* test. The data are shown using Tukey box plots. Values of *P* < 0.05 were considered statistically significant. The actual *P*-values are shown in the graphs.
